# Polyacrylamide/Gel-Based Self-Healing Artificial Tympanic Membrane for Drug Delivery of Otitis Treatment

**DOI:** 10.34133/bmr.0049

**Published:** 2024-07-01

**Authors:** Sujin Kim, Woonhoe Goo, Gul Karima, Jun Ho Lee, Hwan D. Kim

**Affiliations:** ^1^ Department of IT Convergence (Brain Korea Plus 21), Korea National University of Transportation, Chungju, 27469, Republic of Korea.; ^2^ Department of Otorhinolaryngology-Head and Neck Surgery, Seoul National University Hospital, Seoul, 03080, Republic of Korea.; ^3^ Department of Polymer Science and Engineering, Korea National University of Transportation, Chungju, 27469, Republic of Korea.; ^4^ Department of Biomedical Engineering, Korea National University of Transportation, Chungju, 27469, Republic of Korea.

## Abstract

One of the bacterial infections caused by tympanic membrane perforation is otitis media (OM). Middle ear inflammation causes continuous pain and can be accompanied by aftereffects such as facial nerve paralysis if repeated chronically. Therefore, it is necessary to develop an artificial tympanic membrane (TM) that can effectively regenerate the eardrum due to the easy implantation and removal of OM inflammation. In this study, we synthesized hydrogel by mixing gelatin and polyacrylamide. Cefuroxime sodium salt was then incorporated into this hydrogel to both regenerate the TM and treat OM. Cytotoxicity experiments confirmed the biocompatibility of hydrogels equipped with antibiotics, and we conducted drug release and antibacterial experiments to examine continuous drug release. Through experiments, we have verified the excellent biocompatibility, drug release ability, and antibacterial effectiveness of hydrogel. It holds the potential to serve as an effective strategy for treating OM and regenerating TM as a drug delivery substance.

## Introduction

The tympanic membrane (TM) is a thin tissue that separates the outer and middle ears and is one of the essential sensory systems present in the ear [1]. When listening to sound, it vibrates and converts sound waves into nerve stimulation to transmit sound waves to the middle ear [2]. It also acts as a barrier to protect the middle ear from water, bacteria, and other foreign substances. However, complications such as hearing loss and otitis media (OM) can occur if the TM bursts. When tympanic membrane perforation (TMP) occurs, symptoms such as ear pain, bleeding, tinnitus, and nausea caused by dizziness can result in marked challenges in daily life [3]. Furthermore, severe cases can lead to hearing loss [4]. The common causes of TMP include external injury and OM. TMP resulting from external damage is induced due to changes in air pressure in the ear canal and physical contact. TMP caused by OM is inflamed by the penetration of bacteria or viruses into the middle ear [5,6]. In the beginning, acute OM appears, but if the treatment period is missed, it worsens to chronic OM, leading to large TMP that cannot be self-healing, resulting in complications [7]. Treatment requires patching or surgical procedures. However, TMP is one of the diseases that many patients are struggling with because there is a risk of recurrence of OM or reperforation even after surgery [8]. Therefore, it is necessary to develop TMP treatments that can prevent the recurrence and complications of OM.

Native TMs comprise collagen fibers, epithelial cells, and connective tissue, while artificial TMs are fabricated using synthetic materials or biomimetic structures capable of resembling the morphology of the native TM. Native TMs are crucial in acquiring sound energy from the outer ear to generate vibrations. Artificial TMs aim to mimic these functions by providing sound transmission and protection. Regarding biocompatibility, native TMs are naturally biocompatible, but artificial TMs must also exhibit biocompatibility to avoid any adverse reaction or tissue rejection. Native TMs are durable but susceptible to damage and rupture due to the infection or injury. Artificial TMs should withstand normal physiological conditions and show resistance to degradation over time [9,10]

A technique or surgery to avoid perforation is performed to prevent complications caused by infection when TMP occurs [11,12]. Procedures include using patches from materials such as paper and silk to help torn TM regenerate independently. Adding a patch to the perforated TM normalizes the damaged area and helps restore the TM quickly. However, this treatment is only available if the size of the TMP is small [8]. If the TMP lasts 2 to 3 months or there is no possibility of regeneration, surgery is required. In particular, TMP in chronic OM is rare to heal naturally, so surgery is essential. Surgery is performed by artificial TM surgery through tympanoplasty to create a new TM. Artificial TM is smooth and has moderate strength, making it easy to implant and shortening the regeneration period. Therefore, interest in developing artificial TM for effective TM regeneration is increasing [9].

Artificial TM treatments currently being developed include myringoplasty and tympanoplasty [13]. The patient’s temporal fascia restores the TM [14]. Although highly biocompatible, they strain the patient regarding the hassle of performing double surgery. In addition, these surgical methods can cause bleeding, blood clots, pain, and infection during the surgical process because they enter the middle ear by incising an elliptical tympanometry flap of about 8 mm. In addition, there is a limitation in that scars from surgery remain, and the recovery period is extended. Furthermore, implantation of artificial TM without treatment for OM causes recurrence and deterioration of OM, resulting in complications [15]. In conclusion, it is required to develop artificial TM with excellent biocompatibility and can treat inflammation and release drugs to overcome OM.

**Fig. 1.**
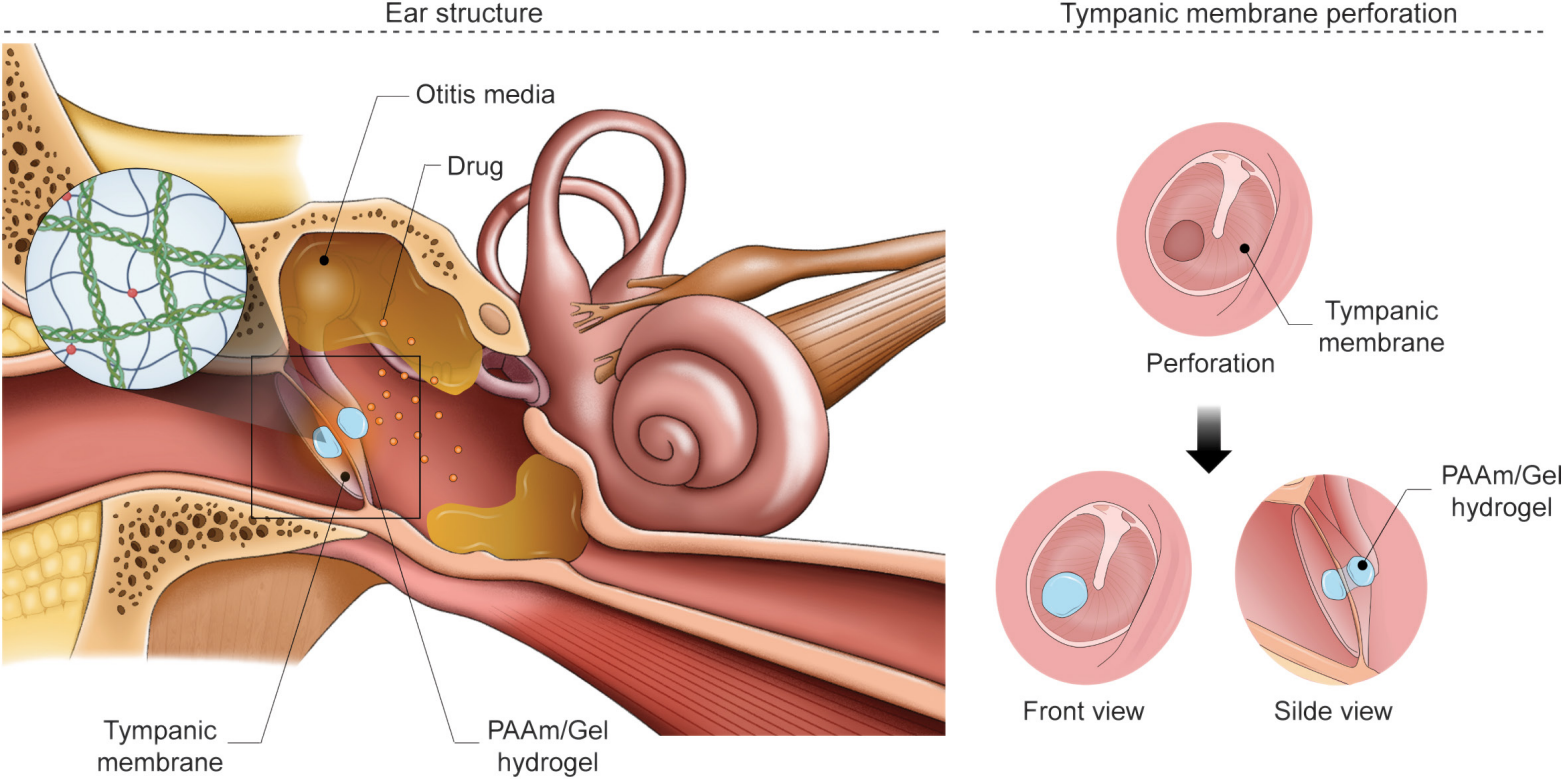
Schematic diagram of the PAAm/Gel@CSS TM with healing and drug delivery during TMP.

In this study, we aim to fabricate a biocompatible artificial TM material that effectively decreases the healing time by incorporating polyacrylamide (PAAm). Artificial TM that is physically and functionally identical to natural TM is made possible by PAAm, a biocompatible polymer. We also explored the potential benefits of PAAm-based artificial TM compared to conventional therapeutic methods. PAAm-based artificial TM has the potential to revolutionize TMP treatment. These implants provide a more effective alternative to conventional surgical methods and can improve the quality of life for millions suffering from TMP. Therefore, by utilizing PAAm with exceptional biocompatibility, we have developed an artificial TM material with strong adherence and the capability to accelerate the healing process. Furthermore, a medication that addresses middle ear inflammation is affixed to the artificial TM to create a multipurpose drug-release artificial TM system that counteracts the aggravation and recurrence of middle ear inflammation while preventing complications (Fig. 1).

## Materials and Methods

### Materials

Acrylamide (AAm) (A8887, China), gelatin from bovine skin type B (G6650, USA), N,N′-methylene bis (acrylamide) (M7279, China), ammonium persulfate (09913, Germany), N,N,N′,N′-tetramethylethylenediamine (T7024, USA), cefuroxime sodium salt (CSS) (C4417, USA), fluorescein 5(6)-isothiocyanate (FITC) (46950, USA), dimethyl sulfoxide (D2650, USA) were purchased from Sigma-Aldrich. Silicone elastomer kits were purchased from 4science (DC-184, Korea). Difco LB Broth, Miller (Luria-Bertani) (244620, USA) was purchased by BD Difco. Agarose Basic was purchased from PanReac ApplyChem (A8963, Italy).

### Fabrication of molds for TM hydrogel

To make a TM-sized hydrogel, a molded mask of width 9 mm, length 8 mm, and thickness 1 mm was manufactured with a 3-dimensional printer. Then, a polydimethylsiloxane (PDMS) solution mixed with silicone elastomer base and silicone elastomer curing agent was produced, poured over the mold mask, removed all air bubbles in the vacuum chamber, and cured in a 65 °C oven for 2 h.

### Synthesis and fabrication of PAAm/Gel hydrogel

Before making the hydrogel, a PAAm solution and a gelatin solution, respectively, were prepared. The PAAm solution was stirred at 60 °C for 20 min with 0.939 g of AAm and 1.878 mg of N,N′-methylene bis (acrylamide) in 6 ml of distilled water. The gelatin solution was made to a 200 mg/ml concentration and dissolved sufficiently at 60 °C. The prepared PAAm and gelatin solutions were mixed in a 19:1 ratio to prepare a PAAm/Gel solution. Then, 15 μl of ammonium persulfate solution and 0.6 μl of N,N,N′,N′-tetramethylethylenediamine were added, placed in a premade mold, and gelled at room temperature overnight.

### Characterization of the PAAm/Gel hydrogel

For scanning electron microscope (SEM, HTACHI, SU3800, Japan) imaging of PAAm/Gel hydrogel, hydrogel samples were frozen at −80 °C for 3 h and freeze-dried overnight in a freeze dryer (Table Top Freeze Dryer, TFD8501, IlShinBioBase, Korea). Platinum sputtering was coated for SEM imaging of the freeze-dried hydrogel sample. ImageJ software (NIH, USA) quantified the pore size of the hydrogel. To measure the swelling ratio (%) of the hydrogel, the dry weight of the freeze-dried hydrogel was measured. The freeze-dried hydrogel was immersed in distilled water and swollen for 0, 30, 60, 180, 300, and 1,440 min. The dry weight was measured by freeze-drying to remove water after swelling. The swelling ratio was calculated using the following equation (Eq. 1):

$$\text{Swelling ratio}\;\left(\%\right)=\frac{\left(W_{s}-W_{d}\right)}{W_{d}}\times 100$$ (1)

W_d_ and W_s_ represent the weight of the dried hydrogel and the hydrogel in the swollen equilibrium state, respectively.

In addition, biodegradation tests of the PAAm/Gel hydrogel were performed. First, the manufactured PAAm/Gel hydrogel was dried in the oven for 30 min and then weighed. The PAAm/Gel was then incubated for 7 and 14 d at 40 °C, 80 rpm after being immersed in phosphate-buffered saline (PBS). After a certain period, the PAAm/Gel hydrogel was dried in an oven for 30 min and then weighed. The degradation rate was calculated using the following equation (Eq. 2):

$$\text{Degradation rate}\;\left(\%\right)=\frac{\left(\mathrm{Wi}-W_{f}\right)}{W_{i}}\times 100$$ (2)

W_i_ and W_f_ represent the initial and late weights of hydrogel, respectively. The average values were reported.

### Tensile strength of hydrogel

To measure tensile strength, adhered samples with adhesion areas of width 0.3 cm and depth 1.2 cm were prepared and tested with a texture analyzer (SurTA 1A, Chemilab Co, Korea). All tests were conducted with a constant tensile speed of 10 mm/min. Tensile strength was determined by dividing the maximum force by the adhesion area.

### Shear strength in porcine skin

Porcine skin was obtained from a local butcher store. To measure shear strength, adhered samples with an adhesion area of the width of 2.5 cm and length of 1 cm were prepared and tested by the standard lap-shear test (ASTM F2255) with a texture analyzer (SurTA 1A, Chemilab Co, Korea). All tests were conducted with a constant tensile speed of 50 mm/min. Shear strength was determined by dividing the maximum force by the adhesion area.

### Synthesis of the PAAm/Gel hydrogel with FITC-gelatin

To check how much gelatin could penetrate through the hydrogel or tissue, FITC-gelatin was synthesized first. One milligram of FITC was dissolved in 0.1 ml of dimethyl sulfoxide to obtain a 10% (w/v) gelatin solution, and the mixture was stored at 60 °C for 1 h. The gelatin solution was placed in a 3.5-kDa M_W_ dialysis tube and dialyzed in distilled water for 5 d to remove unbound FITC. Distilled water was freshly replaced every day for dialysis. On the last day, the FITC-gelatin solution was freeze-dried to synthesize the PAAm/Gel hydrogel with FITC-gelatin under the same protocol as the PAAm/Gel hydrogel synthesis.

### Self-healing test of PAAm/Gel hydrogel with FITC-gelatin

Two groups of hydrogels were synthesized: PAAm/Gel hydrogel with FITC-gelatin and PAAm/Gel hydrogel without FITC-gelatin. The synthesized PAAm/Gel hydrogel with FITC-gelatin was attached to the PAAm/Gel hydrogel without FITC-gelatin. After attachment, hydrogels were stored in the 60 °C chamber for 1 h for self-healing. Then, hydrogels were cooled down to room temperature. Intensity by penetration distance from the contacting area was calculated using Zen Lite (Zeiss, Germany) software.

### Multiple self-healing tensile strength

To measure multiple self-healing tensile strengths, adhered samples with adhesion areas of width 0.3 cm and depth 1.2 cm were prepared and tested with a texture analyzer (SurTA 1A, Chemilab Co, Korea). All tests were conducted with a constant tensile speed of 10 mm/min. Tensile strength was determined by dividing the maximum force by the adhesion area. After the self-healing test, the incised hydrogel was reconnected, stored in a 60 °C chamber for 1 h to self-healing, and then cooled to room temperature to measure tensile strength 4 times.

### Self-healing test of PAAm/Gel hydrogel using infrared fluorescence microscope

To simulate self-healing when PAAm/Gel hydrogel was applied to the TM, a self-healing test was performed using an infrared lamp (HH2500, Philips). The distance between the hydrogel and the infrared was set to 7 to 8 cm, and the temperature was manually adjusted so that the hydrogel remained at 60 °C for 1 h. After that, the hydrogel was cooled at room temperature to confirm self-healing.

### Drug loading into PAAm/Gel hydrogel

PAAm/Gel hydrogel was prepared to load the drug, dried in an oven at 60 °C, and then immersed in 1, 2, and 3 mg/ml of drug solution at room temperature, respectively. Short-term loading was performed to maintain the function and shape of the hydrogel, and the hydrogel weight was measured after loading for 5, 10, 20, and 30 min. The swelling ratio was calculated using the equation (Eq. 1). W_d_ and W_s_ represent the weight of the dried hydrogel and the hydrogel in the swollen equilibrium state, respectively. The loading efficiency was calculated using the following equation (Eq. 3):

$$\text{Drug loading}\;\left(\%\right)=\frac{W_{f}-W_{i}}{W_{i}}\times 100$$ (3)

Here, W_i_ is the hydrogel,s initial weight (mg), and W_f_ is the final weight (mg) of the hydrogel loaded with the drug after drying.

### Drug release ultraviolet-visible and cumulative release (%)

Drug release of PAAm/Gel@CSS was monitored at temperatures of 25 and 37 °C using PBS. Hydrogel loaded with drugs for each concentration was put in 10 ml of PBS and collected in small amounts at 25 and 37 °C. Drug release from the hydrogel of the solution was observed using a microplate reader (Infinite 200 Pro, Tecan, Swiss) at a specific peak of 280 nm. All experiments were carried out in 5 redundancies, and the average value was reported. The cumulative drug release was calculated as follows (Eq. 4):

$$\text{Cumulative release}\;\left(\%\right)=\frac{10C_{n}+\sum 1.2C_{n-1}}{W_{0}}\times 100$$ (4)

*W*_0_ is the weight of the drug in the hydrogel; *C_n_* and *C*_*n*−1_ are the CSS concentrations extracted *n* and *n* − 1 (*n* > 0) times in the buffer solution, respectively.

### Cell culture

Human dermal fibroblasts (HDFs) were obtained from Seoul National University, Korea. In addition, HDFs were cultured in Dulbeco,s modified Eagles medium (#1000136026, STEMCELL, Canada) supplemented with 10% fetal bovine serum (26140079, Gibco, USA) and 1% penicillin–streptomycin–glutamine (10378016, Gibco, USA) to observe the cytotoxicity released from hydrogels. All cells were cultured in a 5% CO_2_ / 95% O_2_ incubator at 37 °C, and fresh media was replenished every 2 to 3 d.

### Live/dead assay

HDF and hydrogel were cocultured to confirm the biocompatibility of PAAm/Gel hydrogel. HDF (5 × 10^4^ cells/well) were seeded in a 12-well plate. In addition, PAAm/Gel hydrogels were placed in the upper chamber of the Transwell insert (SPLInsert Hanging, 37012) and cultured. Briefly, the experimental process was carried out by dyeing HDF cocultured with PAAm/Gel hydrogels using LIVE/DEAD assay (L3224, Invitrogen, USA) on the 1, 3, and 5 d. Images were captured and visualized using a fluorescence microscope with Zen Lite software. Cell viability was calculated by the number of living cells relative to the total number of cells. All experiments were conducted at least 3 times.

### Antibacterial test

*Escherichia coli* (*E. coli*) and *Staphylococcus aureus* (*S. aureus*) were used to detect the antimicrobial capacity of PAAm/Gel hydrogel loaded with CSS through in vitro experiments. *E. coli* and *S. aureus* were obtained from Seoul National University, Korea. Firstly, for preparing agar plates, 0.3 g of agarose and 0.5 g of lysogeny broth were put in a conical flask in 20 ml of distilled water and dissolved by heating in an autoclave (SH-AC-60M, SH SCIENTIFIC). An agar plate was prepared by pouring the solution into a petri dish. A 2.5% lysogeny broth solution was prepared to cultivate *E. coli* and *S. aureus*. Five milliliters of the above solution was extracted and poured into a 14-ml conical tube, and *E. coli* and *S. aureus* were inoculated into the solution, respectively, and then cultured in a shaking incubator at 37 °C, 200 rpm for 24 h. An agar plate was inoculated with 100 μl each of *E. coli* and *S. aureus* cultured for 24 h and spread evenly with a smear stick. The 2 bacteria were diluted with 1 × 10^7^ colony-forming units/ml with distilled water. The PAAm/Gel@CSS prepared by the abovementioned method was placed on an agar plate inoculated with bacteria and cultured in a 37 °C incubator for 24 h. The suppression zone was then quantified. Bacterial counting tests were used to evaluate the antibacterial activity of each sample. The antimicrobial rate was calculated using the following formula (Eq. 5):

$$P\;\left(\%\right)=\left(A-B\right)÷A\times 100\;\left(\%\right)$$ (5)

A represents the average number of bacteria groups in the blank, and B represents the number in another sample.

### Human temporal bone preparation

This study was approved by the Seoul National University College of Medicine/Seoul National University Hospital Institutional Review Board (E-2311-005-1480). A fresh-frozen human temporal bone (TB) without a history of otologic disease was used and acquired from Science Care (Phoenix, Arizona, USA). The TB was extracted from human cadavers within 72 h postmortem. The TB was initially frozen and defrosted 24 h before the test. To maintain moisture and immunity, the TB was immersed in 0.9% saline solution mixed with Betadine (15% Betadine; 85% saline) at 4 °C [16]. All measurements on the bone were performed within 7 d of description.

### Water penetration test on TM

A total of 4 groups were established to test for water invasion in the TM: native TM, perforated, patch shape, and dumbbell shape. The TM was prepared with cuttings along with the surrounding bones to prevent the morphological transformation of the TM. Water infiltration was observed by dropping 100 μl of distilled water on top of both sides of the TM fixed using tongs. In addition, by measuring the amount of water lost downwards, water loss (%) was calculated in the following formula (Eq. 6):

$$\text{Water loss}\;\left(\%\right)=\frac{W_{f}-W_{i}}{W_{d}}\times 100$$ (6)

Here, W_i_ is the initial weight (mg) of the beaker, W_f_ is the final weight (mg) of the beaker that fell down after water penetration, and W_d_ is the mass of water that has been dropped.

### TM movement measurement

The TB with abnormal TM or middle ears was excluded from this study. This measurement was conducted on an isolated vibration table in our laboratory (Biomimetic Material Laboratory) to prevent arbitrary noise. A loudspeaker (ER-2, Etymotic Research, USA) and the microphone probe (ER-7, Etymotic Research, USA) were placed in an artificial external auditory canal (AEAC, 9-mm internal diameter, 8-mm length, <0.5-ml inner volume). The AEAC was mounted and sealed acoustically using epoxy and silicone glue to the cut external auditory canal near the TM rim [17]. A pure tone sound sine wave in the 0.5- to 10-kHz range was stimulated to TM via the loudspeaker. The frequency steps were 500 Hz from 0.5 to 1 kHz and 1 kHz from 1 to 10 kHz. The sound pressure level (SPL) in the AEAC was stimulated at an average of 101-dB SPL (94- to 114-dB SPL) and measured by the microphone probe in front of the TM at approximately 3 mm. A laser Doppler vibrometer (LDV) system (CLV-2534, Polytec GmbH, Germany) was used to calculate the umbo displacement. Displacement of the umbo were measured from the EAC side of the TM with a reflect powder placed on the umbo [18]. VibSoft software (Polytec GmbH, Germany) carried out the stimulation and measurements. TM movement was measured from a laser aimed at the umbo through 0.2-mm thickness antireflective glass (Tova Optec, Korea).

### Immunostaining of the TM

We collected TM portion from TB and performed histopathological study. TM treated as each group was fixed with 3.6% paraformaldehyde (47608, Sigma-Aldrich, Switzerland). In addition, paraffin block and section slide fabrication as well as hematoxylin and eosin (H&E) staining were performed in Celltisbio. The perforation % of stained TM was quantified by calculating the perforation length among the total TM length.

### Statistical analysis

Quantitative results were displayed in the form of mean ± standard deviation. One-way analysis of variance (ANOVA) and 2-way ANOVA of Šídák,s multiple comparisons test were used to identify significant differences between experimental groups. *P < 0.05, **P < 0.01, ***P < 0.001, ****P < 0.0001.

## Results

### Fabrication and characterization of PAAm/Gel hydrogel

TM has mechanical properties such as viscoelastic because it converts sound waves into nerve stimulation. In this study, hydrogel was used to make artificial TM that mimic the properties of TM. Hydrogel is a hydrophilic polymer network that contains water and, at the same time, swells up to a substantial water fraction [19]. Artificial TM was implemented using properties such as chemical functionality, biocompatibility, controllable physical properties, and drug loading capacity of these hydrogels [20]. On the other side, collagen, one of the TM components, is very rich in extracellular matrix, which helps maintain TM,s resilience and plays a role in maintaining mechanical hardness [1]. To mimic this, gelatin was used to synthesize it to crosslink with PAAm network.

**Fig. 2.**
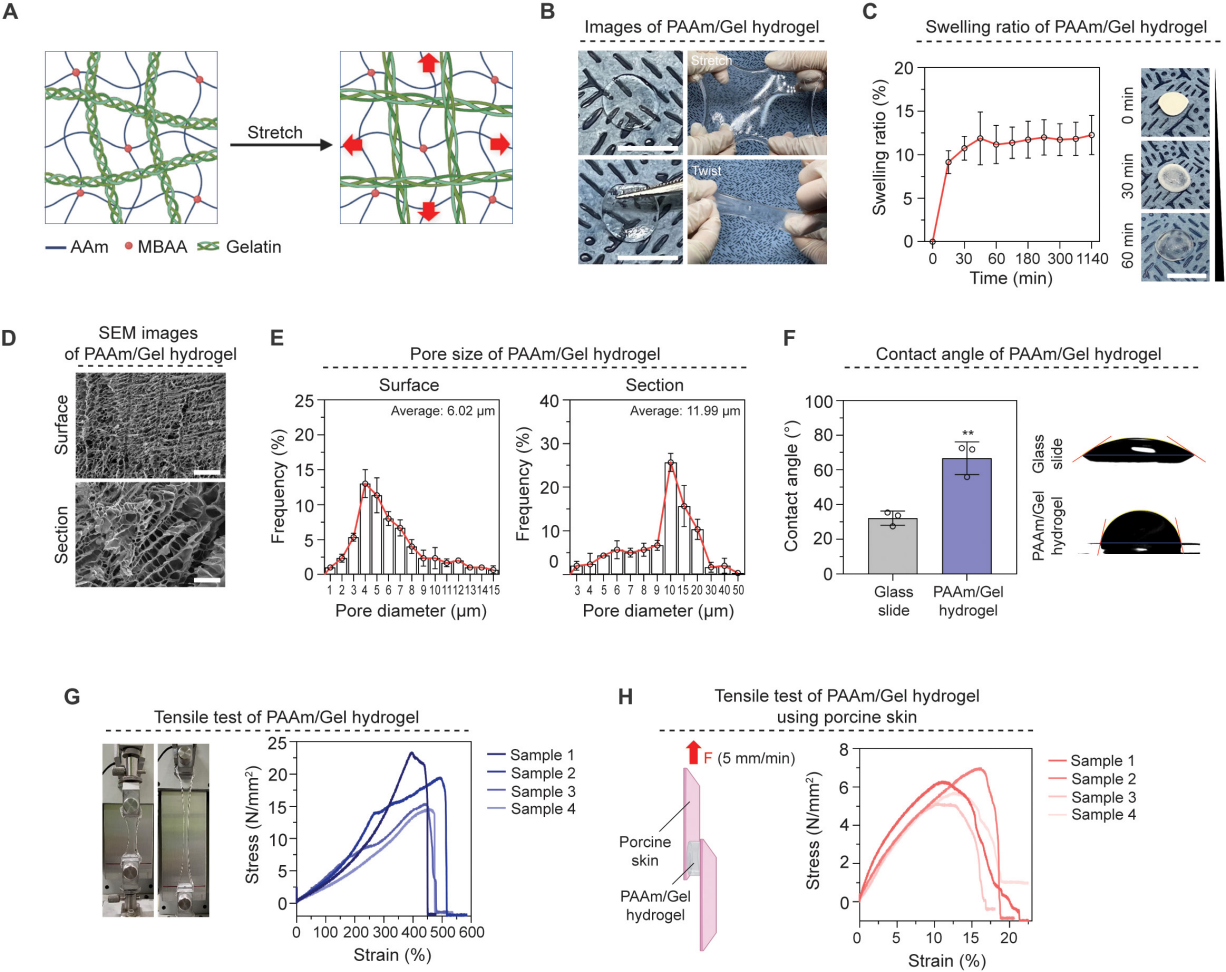
Fabrication and characterization of PAAm/Gel hydrogel. (A) A schematic diagram of the production and characteristics of PAAm/Gel hydrogel. (B) Images of PAAm/Gel hydrogel when stretched and twisted. (C) Swelling ratio of PAAm/Gel hydrogel to time (*n* = 5). (D) SEM images of PAAm/Gel hydrogel (X300). (E) Pore size of the surface and PAAm/Gel hydrogel cross-section (*n* = 3). (F) Graph and images of the contact angle of water on PAAm/Gel hydrogel (*n* = 3). (G) Tensile stress–strain curves representing the elastic efficiency of hydrogels (*n* = 4). In N/mm^2^, “N” represents Newton. (H) Determination of shear strength in porcine skin based on standard lap-shear test (ASTM F2255) (*n* = 4). Scale bars: (B) 10 mm, (C) 10 mm, and (D) 200 μm. All data represent mean ± SD. **P < 0.01. The symbol * indicates comparisons with glass slide.

Before making hydrogel, we used PDMS to create molds that maintain hydrogel shape (Fig. S1). Next, PAAm and gelatin solutions were prepared respectively, then put in a premade PDMS mold and synthesized to make PAAm/Gel hydrogel (Fig. S2). PAAm/Gel hydrogels are crosslinked by covalent bonds between PAAm and gelatin to form a network (Fig. 2A). In particular, due to the tension and contraction of gelatin, hydrogels have remarkable viscoelasticity and elasticity. The manufactured PAAm/Gel hydrogel was transparent and showed a flexible form without tearing when stretched and twisted using hands (Fig. 2B). We further freeze-dried the hydrogel, which was made to measure the swelling ratio of the PAAm/Gel hydrogel. The swelling ratio of hydrogel was calculated by measuring the weight after swelling in PBS for 30, 60, 180, 300, and 1,440 min, respectively. As a result, the PAAm/Gel hydrogel swelled rapidly for 0 to 30 min, and after 30 min, the swelling ratio remained constant and saturated (Fig. 2C). The surface and cross-section of the PAAm/Gel hydrogel were photographed using SEM, respectively. It was confirmed that pores were entirely distributed on both the surface and cross-section of the hydrogel (Fig. 2D). When the distribution by pore size was quantified, the average pore size at the surface and cross-section was measured to be 6.02 and 11.99 μm, respectively (Fig. 2E). One of the characteristics of hydrogel is that its surface is hydrophobic. To confirm its hydrophobicity, the contact angle for water was measured by comparing slide glass with cover glass coated with PAAm/Gel hydrogel. The contact angle was estimated at 32.16° and 66.73° in slide glass without hydrogel coating and cover glass coated with PAAm/Gel hydrogel, showing a difference of twice (Fig. 2F).

Tension is crucial because the TM must tolerate vibration and changes in atmospheric pressure brought on by sound waves. The PAAm/Gel hydrogel’s tensile strength was determined by slowly pulling the hydrogel end after it had been put into the grasp. The hydrogel was cut at approximately 480% strain when 4 samples were evaluated, and the average stress was 18.15 M/mm^2^ (Fig. 2G). For the ease of use and physical attachment of artificial TM, adhesiveness and viscoelasticity are crucial. To test tensile strength, porcine skin, identical to human skin, was chopped into a specified size and linked with hydrogel in between. Consequently, it maintained its unity with an average strain of 19.52% (Fig. 2H and Fig. S3).

### Self-healing properties of the PAAm/Gel hydrogel

**Fig. 3.**
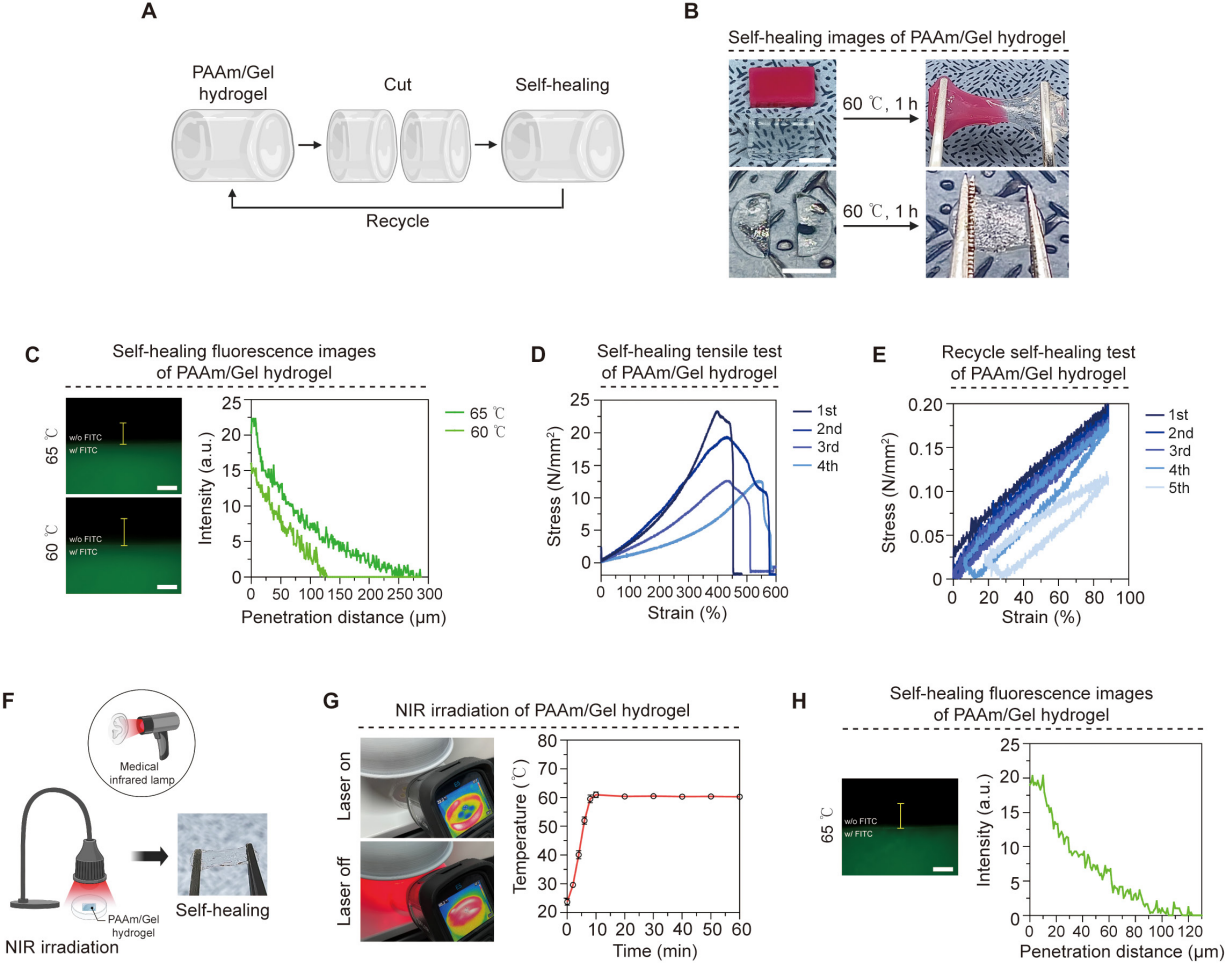
Self-healing properties of the PAAm/Gel hydrogel. (A) A schematic diagram of PAAm/Gel hydrogel self-healing. (B) Recycle self-healing images of PAAm/Gel hydrogel. (C) Fluorescence expression of self-healing PAAm/Gel hydrogel (X200). (D) Tensile stress–strain curve representing the elastic efficiency of the recycle self-healing hydrogel. (E) Tensile stress–strain curve of repeated self-healing cycles (5 times). (F) Self-healing schematic diagram of PAAm/Gel hydrogel using medical infrared lamp. (G) Temperature change graph of PAAm/Gel hydrogel by medical infrared lamp (manually control on and off). (H) Fluorescence expression of PAAm/Gel hydrogel by medical infrared lamp. Scale bars: (B) 20 mm, (C) 60 μm, and (H) 60 μm (*n* = 3).

To develop the best implantable artificial TMs, mechanically robust hydrogels can quickly improve defects in materials [21]. We evaluated the TMP of PAAm/Gel hydrogel’s capacity to mend flaws and withstand reperforation. In order to see the self-healing ability of hydrogels, the prepared hydrogel was cut and then crosslinked again. The PAAm/Gel hydrogel was crosslinked at 60 °C for 1 h using gelatin having thermal and rheological properties (Fig. 3A) [22]. Consequently, it was observed that it exhibited flexibility and stretched when pulled with tweezers. A hydrogel was attached to each one (Fig. 3B and Movie S1). To verify whether the interface of the hydrogel is chemically bonded, hydrogels including general PAAm/Gel hydrogel and FITC were prepared, respectively, and then self-healing was performed at 60 and 65 °C. As a result, depending on the PAAm/Gel hydrogel interface, fluorescence was expressed up to approximately 130 μm. In contrast to the 60 °C group, the 65 °C group was measured to have a double width, and the fluorescence range was expressed up to approximately 280 μm (Fig. 3C). To measure the physical strength of the self-healed PAAm/Gel hydrogel, self-healing tests were performed repeatedly through several cycles. As a result of measuring the tensile strength of the hydrogel that has been performed in several processes, the tensile strength tends to decrease as the number of self-healing increases. However, the average tensile strength of the 4 repetitive experiments was 16.97 N/mm^2^, maintaining constant stability (Fig. 3D). In addition, as a result of repeating about 90% strain on the self-healed PAAm/Gel hydrogel 5 times, the stress returned to its original form without breaking or deforming the hydrogel even after stretching and confirmed the resilience by elasticity (Fig. 3E).

Clinics such as otolaryngology often use medical infrared lamps to transfer heat deep into the skin [23]. We similarly conducted a self-healing test with medical infrared lamps to demonstrate the availability of PAAm/Gel hydrogel in clinical treatment conditions (Fig. 3F). After cutting the PAAm/Gel hydrogel, temperature was applied to the hydrogel using a medical infrared lamp. The distance between the lamp and the hydrogel was 7 to 8 cm. The temperature of the hydrogel was controlled by manually turning on and off the lamp and maintaining a constant temperature of 60 to 65 °C (Fig. 3G). After self-healing of hydrogels with and without FITC using the medical infrared lamp, the range of FITC expression at the hydrogel interface was measured. Fluorescence according to the PAAm/Gel hydrogel interface was expressed at a distance of about 120 μm, and it can be seen that not only physical self-healing but also self-healing through chemical bonding occurred. Therefore, the use of infrared lamps used for clinical treatment also showed the possibility of self-healing (Fig. 3H).

### Characterization of PAAm/Gel@CSS

CSS is a second-generation cephalosporin antibiotic that destroys the cell wall and prevents bacterial growth. CSS was introduced as an antibiotic in PAAm/Gel hydrogel to treat and prevent OM as well as the mechanical properties of artificial TM. Before loading the drug into the hydrogel, rhodamine B, a chemical with a similar structure, was used to show the possibility of loading (Fig. S4). Then, PAAm/Gel hydrogel was dried overnight in an oven at 60 °C, and each of PAAm/Gel hydrogel was loaded by immersing them in CSS solution with concentration of 1, 2, and 3 mg/ml. In addition, in order to maintain the adhesion of hydrogel and the internal network structure, the swelling process was predetermined time of 30 min. Consequently, drug-loaded PAAm/Gel@CSS was constructed.

**Fig. 4.**
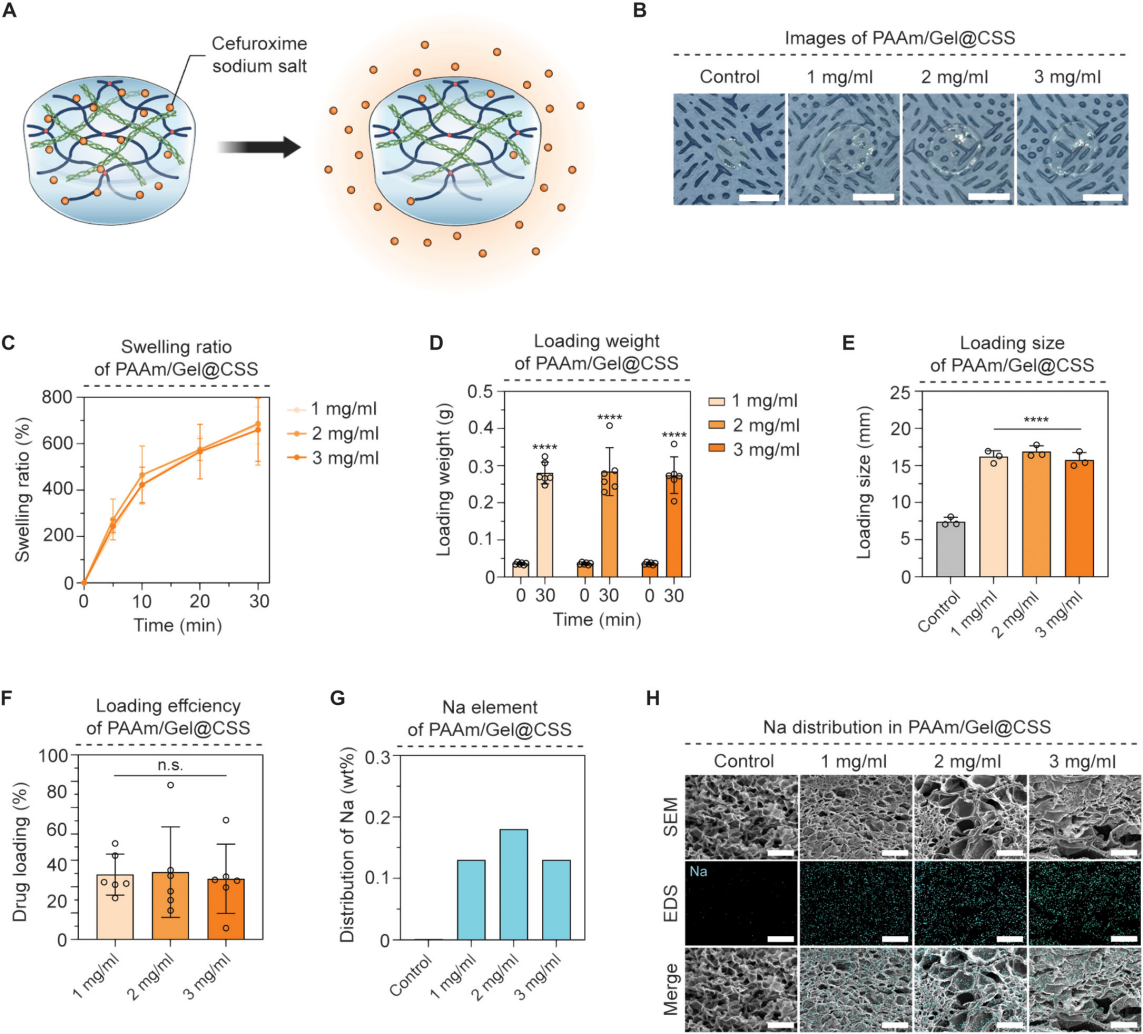
Characterization of PAAm/Gel@CSS. (A) A schematic diagram of PAAm/Gel hydrogel and CSS drug loading. (B) Images of hydrogels before and after drug loaded by concentration (Control: 0 mg/ml). (C) Hydrogel swelling ratio (%) graph by drug concentration (*n* =3). (D) A graph of the loading weight of the hydrogel by drug concentration after 30 min of loading (*n* = 4). (E) Graph of loading size of hydrogel according to drug concentration (*n* = 3). (F) Drug loading (%) graph of hydrogel by drug concentration (*n* = 5). (G) Distribution of sodium elements in hydrogel according to drug concentration. (H) SEM and EDS mapping images of the hydrogel according to drug concentrations. Scale bars: (B) 10 mm and (H) 100 μm. All data represent mean ± SD. ****P < 0.0001. The symbol * indicates comparisons with control (0 mg/ml).

PAAm/Gel hydrogels with a 3-dimensional mesh structure can mount drugs inside the gel through the immersion process in the CSS solution, and CSS mounted inside the hydrogel after swelling in the CSS solution is delivered to a specific area through the process of spreading from high concentration to low concentration (Fig. 4A). Even after drug loading, PAAm/Gel@CSS was transparent (Fig. 4B). The swelling ratio of PAAm/Gel hydrogel in CSS solution of 30 min was measured, and the concentrations of 1, 2, and 3 mg/ml were similar, with an average of 679.27%, 686.89%, and 660.57% respectively (Fig. 4C). In addition, before and after loading, the weight (g) of PAAm/Gel@CSS was measured. After loading, the weight of PAAm/Gel@CSS was approximately 8-fold higher in all groups compared to the weight of the dry hydrogel before loading and the weight was 0.280, 0.284, and 0.275 g for each drug concentration of 1, 2, and 3 mg/ml, respectively (Fig. 4D). In addition, as a result of measuring and comparing hydrogel size, drug concentrations of 1, 2, and 3 mg/ml were measured at 2.15, 2.29, and 2.16 mm, respectively, and PAAm/Gel@CSS doubled at all concentration groups when compared to the control group without CSS (Fig. 4E). The loading efficiency of PAAm/Gel@CSS was derived from the hydrogel weight and size change values obtained before and after drug loading, and depending on the drug concentration, the loading efficiency values were 49.23% in the 1 mg/ml group, 47.77% in the 2 mg/ml group, and 46.04% in the 3 mg/ml group (Fig. 4F).

Additionally, EDS was used to distribution of specific elements to view drug in PAAm/Gel@CSS. When the amount of sodium, an element that appears in CSS, was measured, sodium was 0.13, 0.18, and 0.13 wt% at concentration of 1, 2, and 3 mg/ml, respectively, and sodium distribution did not appear in the control group (Fig. 4G). In addition, the sodium distribution in PAAm/Gel@CSS was mapped and compared with SEM images. Similarly, sodium did not appear in the control group, and sodium was uniformly distributed throughout the hydrogel in all 3 groups loaded with the drug (Fig. 4H). This resulted in successful drug loading with the immersion of CSS into PAAm/Gel hydrogel.

Additionally, we performed biodegradation tests of the PAAm/Gel hydrogel. The PAAm/Gel hydrogel was immersed in PBS for 0, 7, and 14 d, respectively. In addition, we calculated the degradation %. D7 and D14 were measured on average 22.18% and 53.56%, respectively, and D14 was 2.4 times higher than D7 (Fig. S5). Consequently, it indicates that hydrogel biodegrades concurrently with drug release.

### Drug release and antibacterial ability of PAAm/Gel@CSS

**Fig. 5.**
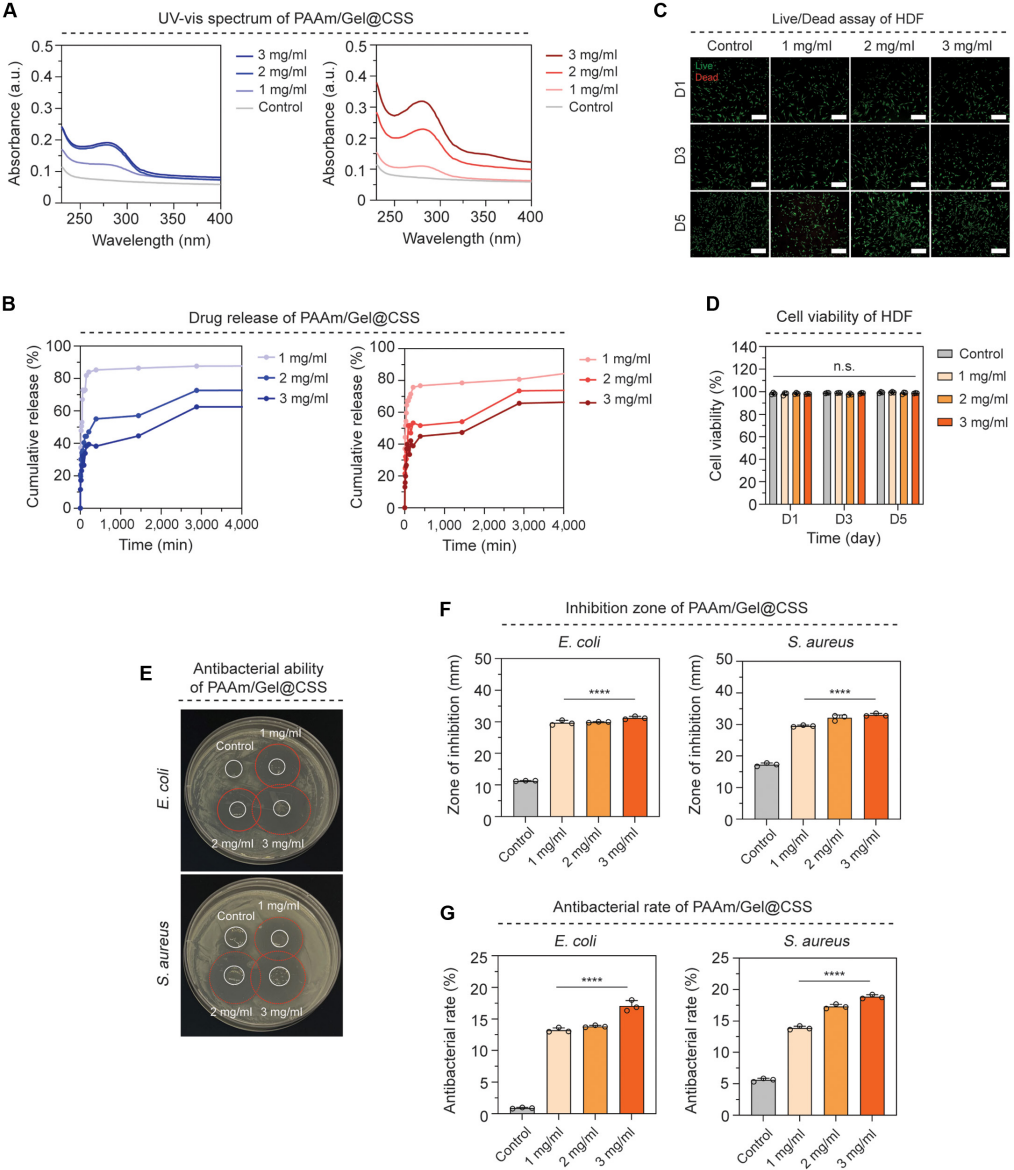
Drug release and antibacterial ability of PAAm/Gel@CSS. (A) Drug release ultraviolet-visible graphs for 4 d of PAAm/Gel@CSS by concentration (Control: PBS). (B) Graph of cumulative release of hydrogel according to drug concentration. (C) Live/dead images (X200) of HDF cocultured with hydrogels loaded by drug concentration for 1, 3, and 5 d (green: live cells, red: dead cells). (D) Cell viability quantification graph. (E) Antimicrobial activity images of hydrogel loaded by drug concentration (*E. coli*, *S. aureus*). (F) Quantification of *E. coli* and *S. aureus* growth inhibition zone of by drug loading concentration. (G) Antibacterial rate (%) graph of hydrogel by drug loading concentration. Scale bar: (C) 200 μm. All data represent mean ± SD (*n* = 3). ****P < 0.0001. The symbol * indicates comparisons with control (0 mg/ml).

Hydrogel functions as a drug carrier and has a specific continuous release effect on the drug. PAAm/Gel@CSS with porous structures are drug delivery system that can release antibiotics to inflammatory area while maintaining their original tissue condition with excellent biocompatibility, and continuous drug release ability [24]. To evaluate the drug-releasing ability of the PAAm/Gel@CSS according to external temperature, experiments were conducted by dividing into groups of 25 °C at room temperature and 37 °C at body temperature. PAAm/Gel@CSS was immersed in PBS for 4,000 min in an environment of 25 and 37 °C. The amount of drug released from PAAm/Gel@CSS was confirmed by absorbance at 280 nm, the specific wavelength band of CSS. In both the 25 and 37 °C groups, the absorbance tended to increase as the drug loading concentration increases. In particular, the absorbance at 37 °C group showed 1.7 times higher for 1 mg/ml, 1.2 times higher for 2 mg/ml, and 0.9 times higher for 3 mg/ml than for 25 °C, indicating a higher drug release (Fig. 5A). The cumulative release of CSS over time was calculated based on the absorbance of CSS. The cumulative release of drugs in the 25 and 37 °C environments was similar, but there was a difference in the release amount depending on the concentration of the drug. At 25 °C temperature, 87.8%, 72.8%, and 62.6%, respectively, depending on the drug concentration of 1, 2, and 3 mg/ml, were found, and at 37 °C temperature, 85.4%, 73.9%, and 66.6%, respectively. As the drug concentration increased, the cumulative release tended to decrease (Fig. 5B). In addition, the results of the 4,000-min data show continuous drug release over time.

Fibroblasts are thought to be the main cell type of skin tissue. Fibroblasts are essential for regenerating and remodeling damaged skin tissue by producing collagen. In this study, to test the biocompatibility of PAAm/Gel@CSS, we performed a coculture with HDFs. HDF cocultured with PAAm/Gel@CSS were live/dead-stained on days 1, 3, and 5, respectively. Depending on the number of days, it was shown that cells grew without much cell debris (Fig. 5C). In the results of quantifying cell viability, cell viability was all measured to be 80% or more without any difference in drug concentration on days 1, 3, and 5, indicating the biocompatibility of PAAm/Gel@CSS (Fig. 5D). To evaluate the antimicrobial capacity of PAAm/Gel@CSS, disk diffusion was used to identify areas of *E. coli* and *S. aureus* inhibition zones depending on drug concentration. In both *E. coli* and *S. aureus*, the PAAm/Gel@CSS group showed a marked area of bacterial inhibition compared to the non-drug-loaded control group, and the antibacterial ability by drug release was shown (Fig. 5E). In the quantification of *E. coli* and *S. aureus* bacterial inhibition zone, the drug conocentrations of 1, 2, and 3 mg/ml groups of PAAm/Gel@CSS were all about 3 times higher than those of the control group (Fig. 5F). Antibacterial rate of each group was calculated by the number of inhibitory strains against *E. coli* and *S. aureus*, of PAAm/Gel@CSS loaded with CSS by concentration. The antibacterial rates of PAAm/Gel@CSS against *E. coli* were 13.2%, 13.8%, and 17.1%, respectively, for each drug concentration of 1, 2, and 3 mg/ml, and the antibacterial rates against *S. aureus* were 13.9%, 17.3%, and 18.9%. PAAm/Gel@CSS showed an increase in antibacterial rates of about 16 times in *E. coli* and about 3 times in *S. aureus*, respectively, compared to control group, displaying excellent antimicrobial capacity (Fig. 5G).

### Insertion ability test of PAAm/Gel hydrogel in TB

**Fig. 6.**
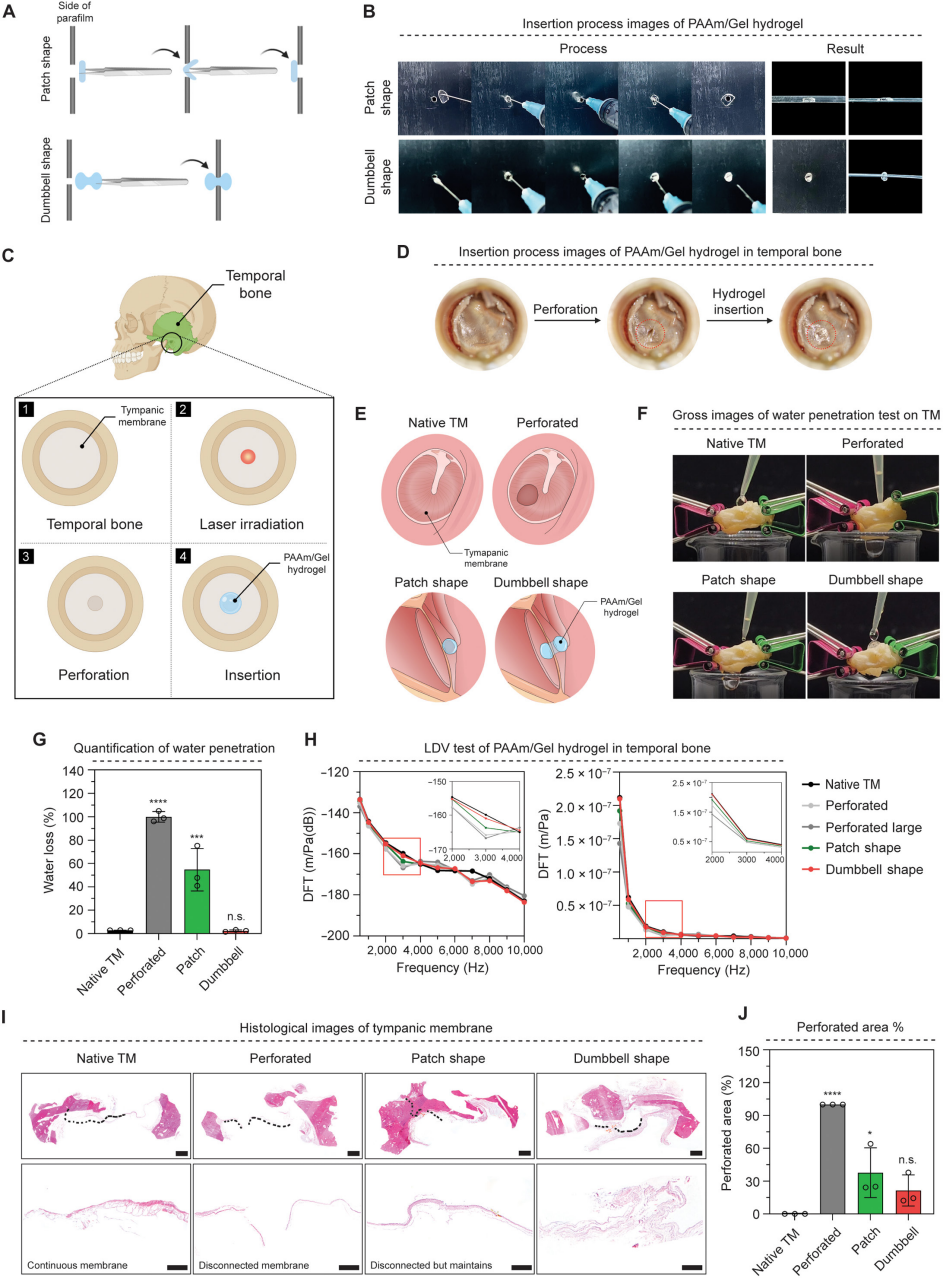
Insertion ability test of PAAm/Gel hydrogel in TB. (A) A schematic diagram of PAAm/Gel hydrogel insertion according to shape. (B) Images of PAAm/Gel hydrogel insertion process (hole size: 2 mm). (C) A schematic diagram of the PAAm/Gel hydrogel insertion process after induction of TMP in TB. (D) Images of PAAm/Gel hydrogel insertion in TB. (E) A schematic diagram of experimental group on PAAm/Gel hydrogel transplantation. (F) Gross images of water penetration test on TM. (G) Quantification of water penetration. (H) DFT graph by PAAm/Gel hydrogel presence and shape. (I) Histological H&E staining image of TM. (J) Quantification of perforation area in TM. Scale bars: (I) 2 mm (above) and 400 μm (below). All data represent mean ± SD (*n* = 3). *P < 0.05, ***P < 0.05, ****P < 0.0001. The symbol * indicates comparisons with native TM.

Artificial TMs must be able to fill the perforation and be inserted to treat TMP. To test the implantability of TM, we separated PAAm/Gel hydrogel into patchy and dumbbell types. A fixed parafilm was punctured to replicate the TMP scenario, and various shaped hydrogels were inserted (Fig. 6A). As demonstrated in the transplant process image, the perforation was filled, and the dumbbell type was fixed. The patch type, on the other hand, was only affixed to one side of the TM (Fig. 6B and Movie S2).

The LDV test was conducted in response to the sound to determine how well the implanted PAAm/Gel hydrogel could withstand vibration. Prior to the LDV experiment, we implanted a shaped PAAm/Gel hydrogel and caused TMP in the human TB (Fig. 6C and D, Fig. S6, and Movie S3). The experimental group was divided into a total of 4 groups: a control group without perforation, a perforation group, a patch shape hydrogel, and a dumbbell shape hydrogel group (Fig. 6E). To ensure that the hydrogels were filling the holes well in each group, water penetration experiments were conducted. When 100 μl of water was dropped over the TM, the loss of water was measured how much water passed through (Fig. 6F and Movie S4). In each group, water loss % was 36- and 20-fold higher than native TM in perforated and patch shape groups, respectively. However, the dumbbell shape group confirmed that water loss % was similar to native TM, and the dumbbell shape hydrogel effectively filled the perforation (Fig. 6G).

To compare the TMP-induced TM with the hydrogel implanted for each shape, the LDV test was carried out. The discrete Fourier transform (DFT) value decreased when TMP occurred, and more specifically, the larger the size of TMP, the lower the value was observed when comparing the native TM and TMP groups. The perforation was steadily filled in the PAAm/Gel hydrogel-implanted group instead of the TMP group because of a rise in DFT. Furthermore, the outcomes of PAAm/Gel hydrogel implants, both dumbbell- and patch-type, demonstrated that the dumbbell type’s curve resembled the control group,s more (Fig. 6H). In addition, H&E staining was performed to observe the PAAm/Gel hydrogel inserted into TM histologically (Fig. 6I). Native TM formed a parallel side without perforation, and the perforation group showed the form of a noncontinuous line segment in the perforation. The patch group and dumbbell group showed a stable insertion into the hydrogel insertion site. Based on this, the perforated area was quantified (Fig. 6J). When native TM was set to 0% and the perforated group was 100%, the patch group was 37.71% and the dumbbell group was 21.45%. Therefore, the dumbbell group showed a perforated area % 0.5 times lower than the patch group, which was similar to native TM, showing effective support for the perforation site.

## Discussion

This study aimed to establish an artificial TM drug release system equipped with a drug capable of treating OM. Integration of CSS into PAAm/Gel hydrogel formulations is a favorable strategy for treating OM due to compensation for defective tissue and continuous drug delivery to the local area [25]. The PAAm/Gel hydrogel includes PAAm and gelatin. PAAm is a polymer with a linear chain structure, and when water is supplied, it forms a soft gel and has high moisture absorption. Gelatin is soft and elastic and has viscoelasticity and self-healing properties due to the crosslinked structure in the hydrogel. In addition, the PAAm/Gel hydrogel is transparent so that sound waves can pass through without distortion, and its crosslinked structure shows excellent swelling [26]. In addition, due to the nature of hydrogels, they can have various forms depending on the mold they are manufactured. As a result, they can be easily molded in various cases, which is suitable for individuals. The excellent biocompatibility and adhesion of PAAm/Gel hydrogels have demonstrated their function as tissue substitutes [27]. They suggest the potential for organ stability with a low risk of dropout after transplantation. The development of PAAm/Gel is marked from material science perspective due to the reasons such as advanced mechanical, self-healing and tunable properties, fatigue resistance, biocompatibility, and biodegradability. Mixing gelatin into PAAm-based hydrogels can markedly enhance the mechanical strength and toughness of the material. The resulting PAAm/Gel double-network hydrogels have exhibited high compressive stress (up to 0.268 MPa) and modulus (up to 84 kPa), outperforming single-network gels. PAAm/Gel hydrogels can showcase rapid self-recovery properties, with up to 87% toughness recovery at room temperatures. This self-healing ability is attributed to the effective energy dissipation through the rupture of the physically crosslinked gelatin network. The combination of high strength and self-healing makes these hydrogels promising for applications requiring durability and damage tolerance. The mechanical and self-healing properties of PAAm/Gel hydrogels can be tailored by adjusting the ratio of the 2 components, the degree of gelatin modification, and the crosslinking density. Gelatin is a natural, biocompatible, and biodegradable polymer, which can impart these desirable properties to the PAAm/Gel hydrogels, expanding their potential for biomedical applications such as wound dressings and tissue engineering scaffolds [28–31].

A temperature of 60 °C was selected to accelerate the self-healing temperature of the PAAm/Gel hydrogel. In addition, Yan et al*.* [28] performed a self-healing test on the PAAm/Gel hydrogel at temperatures ranging from 15 to 60 °C and measured its resilience in 80% of 30 °C or higher. This emphasizes the importance of proper temperature control for clinical applications. Second, the self-healing test group focused on evaluating the unique material properties of the PAAm/Gel hydrogel. Since the self-healing mechanism mainly involves the recrosslinking of PAAm and gelatin, we performed the test on the PAAm/Gel hydrogel without drug load in anticipation of similar results as the PAAm/Gel@CSS group.

The loading capacity of PAAm/Gel@CSS by drug concentration showed high efficiency, and it can be expected to control the release rate through concentration control through cumulative release difference according to drug concentration. Furthermore, suppression of inflammation, treatment of OM, and prevention of complications can also be expected [32]. The antibacterial activity of PAAm/Gel@CSS was assessed using drug release studies. PAAm/Gel@CSS efficiently inhibited bacterial growth, which is the most important aspect in OM treatment. Bacterial infection interferes with the TM regeneration process, and PAAm/Gel@CSS inhibits bacterial growth at the TMP site, helping to treat OM. In addition, excellent biocompatibility allows safe application to the perforation site. The combination of drug-induced antibacterial function in the drug-releasing system of PAAm/Gel@CSS and the alternative function of TM tissue as artificial TM offers many advantages over previous TM transplants. In biocompatibility assessment, cellular viability studies of HDF confirmed the safety of PAAm/Gel@CSS. The PAAm/Gel hydrogel showed excellent biocompatibility in this evaluation and presented a favorable profile for animal experimental application.

Some challenges need to be addressed in future research. Detailed studies on the PAAm/Gel@CSS hydrogel are needed. The drug loading process that preserves the properties of the hydrogel as much as possible should be optimized by controlling it. In addition, assessing the therapeutic efficacy and long-term stability of PAAm/Gel@CSS in vivo could provide additional insights into its in vivo activity and drug release capabilities. Furthermore, it is ultimately necessary to demonstrate its applicability as an artificial TM compared to the materials used in existing treatments. In this study, a multifunctional system that can remove drugs while serving as a mechanically and physically supportive artificial TM and native TM by filling perforations is proposed, highlighting the promising potential of drug-loaded PAAm/Gel@CSS systems to treat OM and regenerate TM. The combination of physical properties and drug delivery of PAAm/Gel hydrogels suitable for these tissues provides a promising way to overcome the limitations of conventional drug delivery systems. PAAm/Gel@CSS has demonstrated efficient and continuous drug delivery, excellent durability and mechanical properties, excellent antimicrobial ability, and biocompatibility, so it has potential as an effective tissue engineering and drug delivery treatment in continuous drug release treatment.

## Data Availability

The data are freely available upon request.
