# Supplementary figures and images for: Polyacrylamide/Gel-Based Self-Healing Artificial Tympanic Membrane for Drug Delivery of Otitis Treatment
Source: Biomater Res. 2024 Jul 1;28:0049. doi: 10.34133/bmr.0049 (PMC11214819; doi:10.34133/bmr.0049)

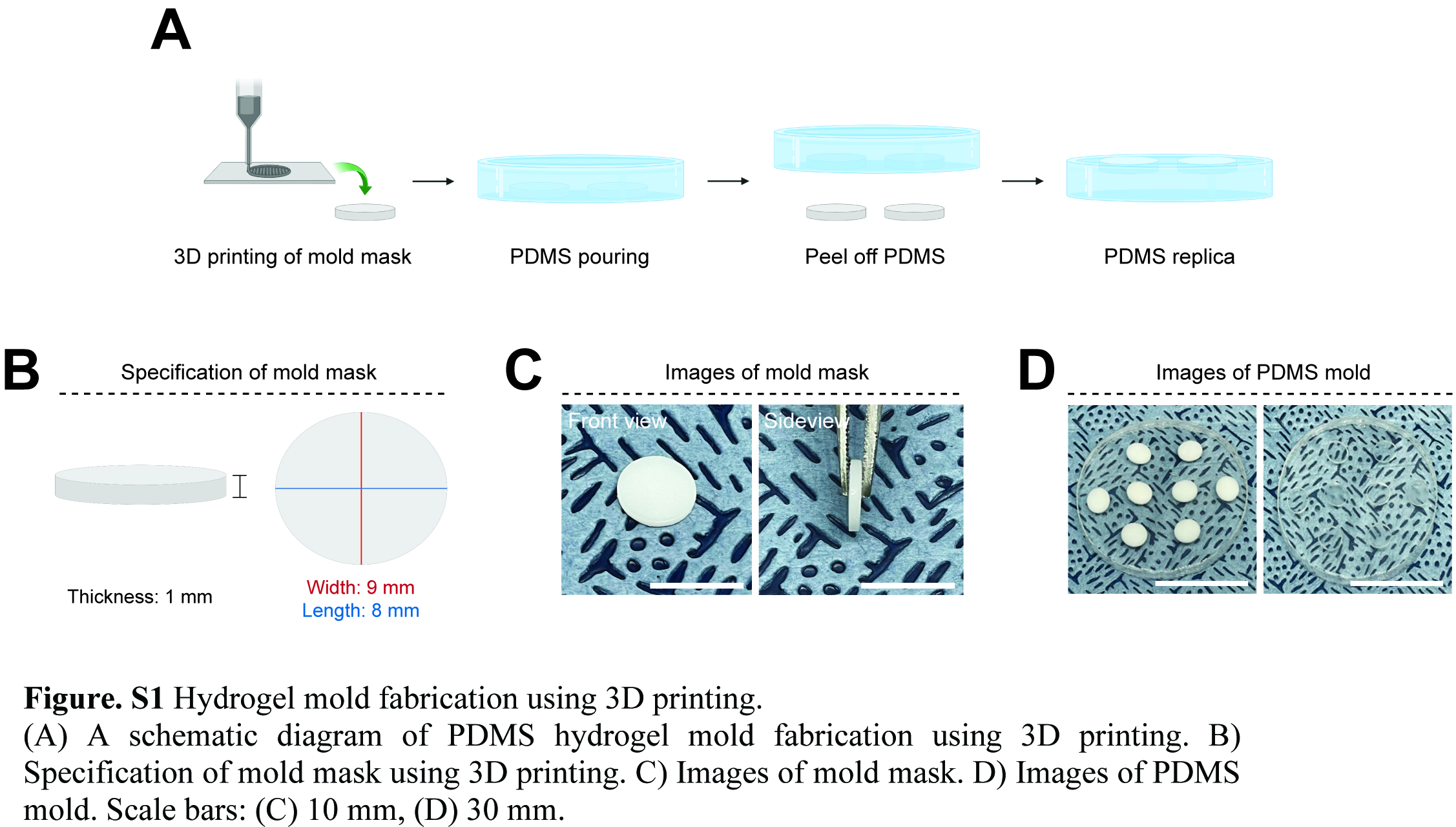

Supplement: Supplementary 1 — Figs. S1 to S6 Movies S1 to S4 [file bmr.0049.f1.zip › Fig S1.tif]

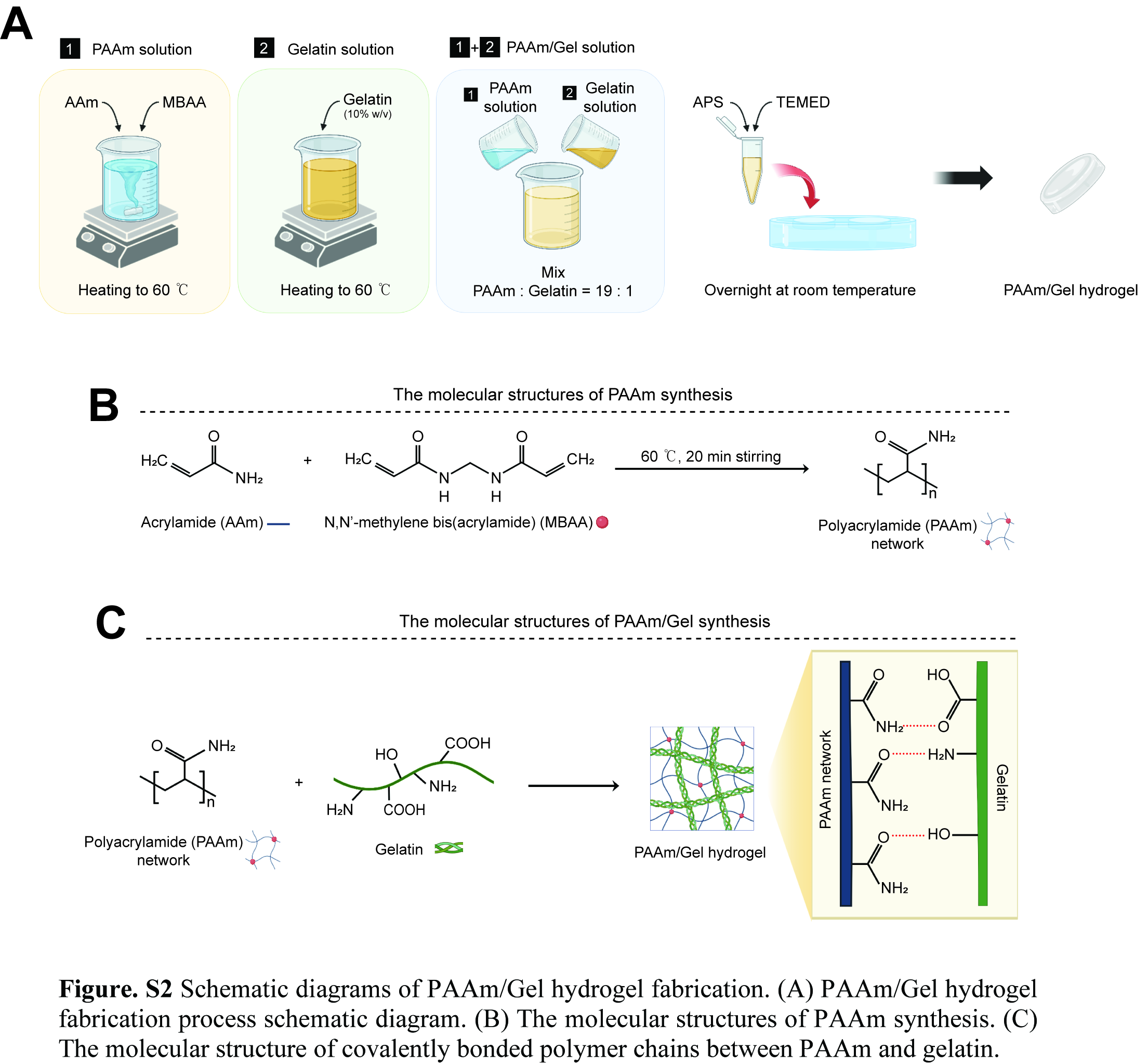

Supplement: Supplementary 1 — Figs. S1 to S6 Movies S1 to S4 [file bmr.0049.f1.zip › Fig S2.tif]

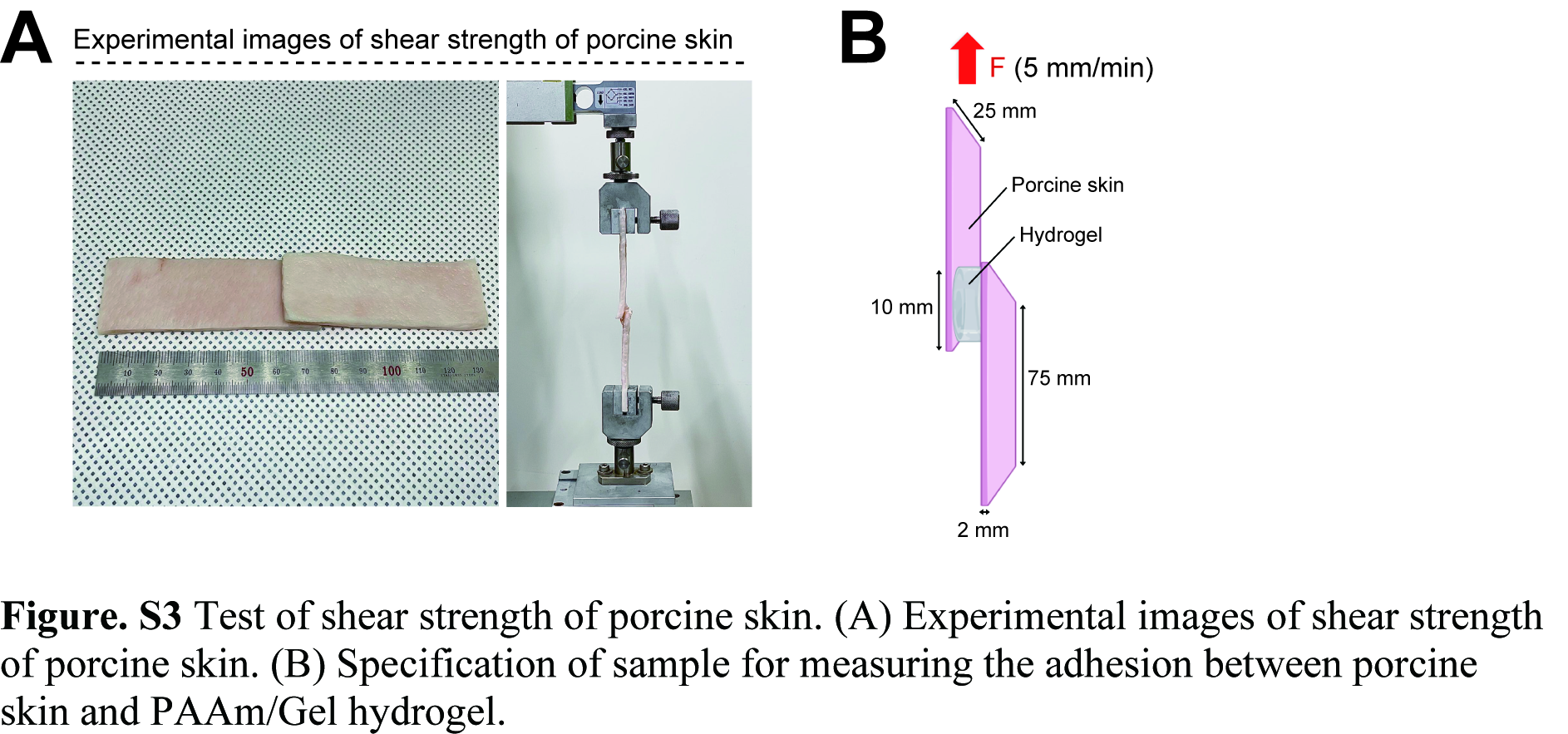

Supplement: Supplementary 1 — Figs. S1 to S6 Movies S1 to S4 [file bmr.0049.f1.zip › Fig S3.tif]

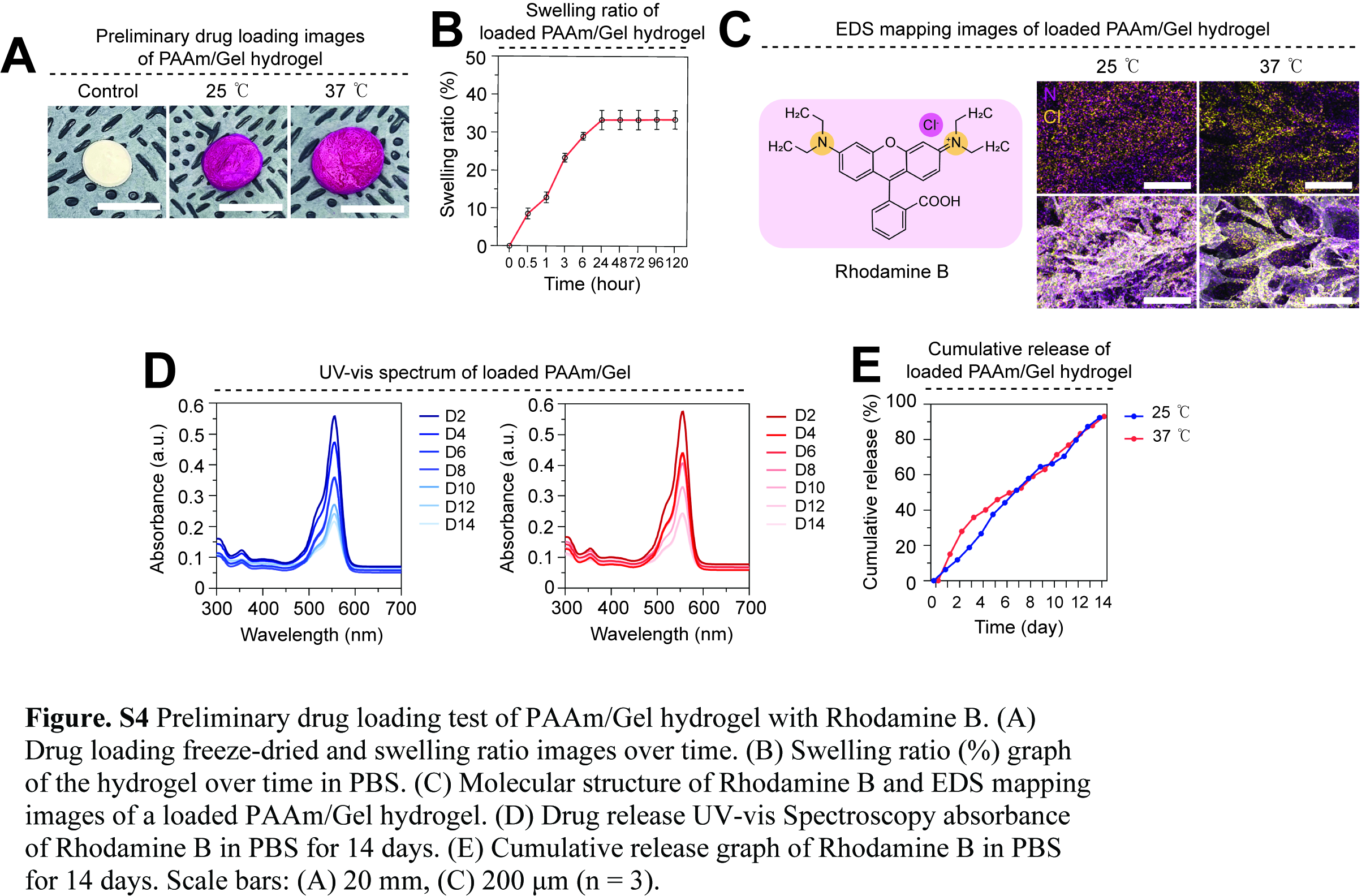

Supplement: Supplementary 1 — Figs. S1 to S6 Movies S1 to S4 [file bmr.0049.f1.zip › Fig S4.tif]

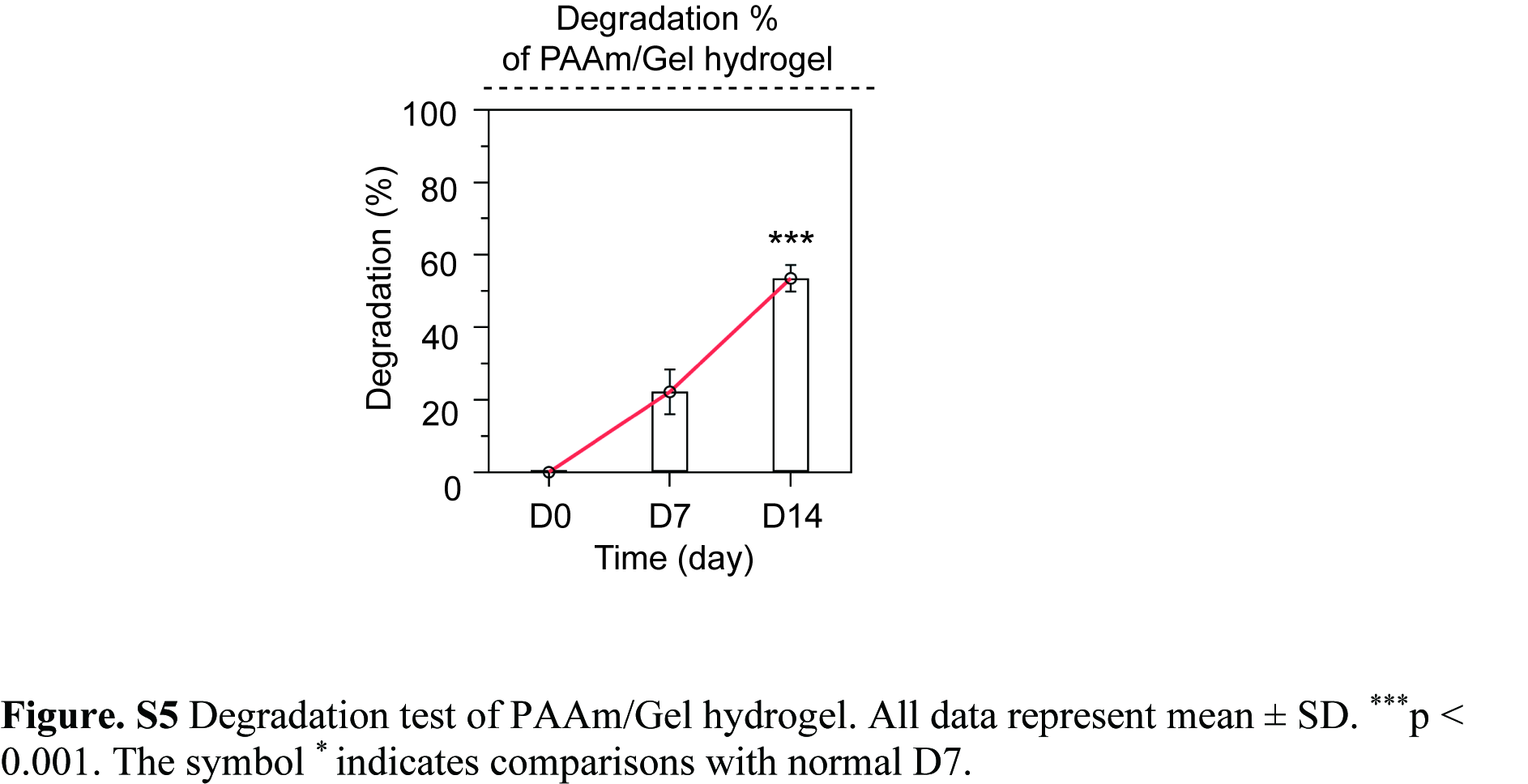

Supplement: Supplementary 1 — Figs. S1 to S6 Movies S1 to S4 [file bmr.0049.f1.zip › Fig S5.tif]

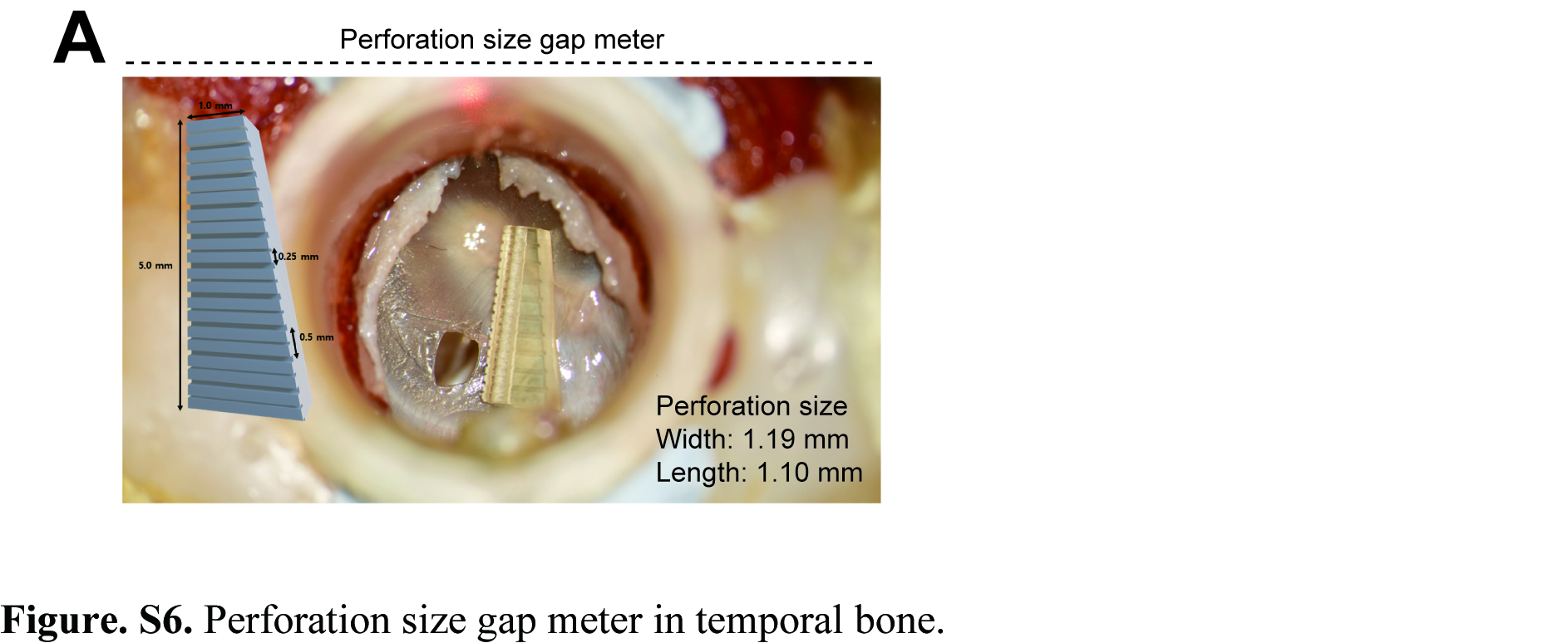

Supplement: Supplementary 1 — Figs. S1 to S6 Movies S1 to S4 [file bmr.0049.f1.zip › Fig S6.tif]
